# The Association of XRCC1 Gene Polymorphisms and Chronic Hepatitis C Induced Insulin Resistance in Egyptian Patients

**DOI:** 10.3390/cells7110185

**Published:** 2018-10-25

**Authors:** Salwa M. Abo El-khair, Mona Arafa, Tarek Besheer, Ahmed M. El-Eraky, Ayman Z. Elsamanoudy

**Affiliations:** 1 Department of Medical Biochemistry and Molecular biology, Faculty of Medicine, Mansoura University, Mansoura 35516, Egypt; ayman.elsamanoudy@gmail.com; 2 Department of Tropical medicine, Faculty of Medicine, Mansoura University, Mansoura 35516, Egypt; monaarafa278@yahoo.com (M.A.); tarekbesheer@yahoo.com (T.B.); omtyomty2@yahoo.com (A.M.E.-E.); 3 Department of Clinical Biochemistry, Faculty of Medicine, King Abdulaziz University, Jeddah 21589, Saudi Arabia

**Keywords:** *XRCC1* gene, polymorphisms, insulin resistance, HCV

## Abstract

Chronic hepatitis C is implicated in insulin resistance (IR) susceptibility. An X-ray repair cross-complementing group 1 gene (*XRCC1*) is proposed to be a candidate gene for a study of IR susceptibility. So, this study aims to investigate the possible association of the *XRCC1* gene polymorphisms with the risk of IR related to chronic hepatitis C virus (HCV) infection in Egyptian patients. In a case-control study, a total of 210 subjects, including 140 chronic HCV patients (87 patients with IR and 53 without IR) and 70 healthy controls, were included. Two genetic polymorphisms (c.1254C > T and c.1517G > C) of the *XRCC1* gene were genotyped via the PCR-restriction fragment length polymorphism (PCR-RFLP) technique. The result of the current study revealed that these two single nucleotide polymorphisms (SNPs) have statistically significant influences on susceptibility to IR in chronic HCV infected Egyptian patients. It could be concluded that c.1254C > T, the TT genotype, CT/CC carriers as well as c.1517G > C, the CC genotype and GC/GG carriers might be associated with increased IR susceptibility. Moreover, T-allele of c.1254C > T and the C-allele of c.1517G > C genetic variants might influence the susceptibility.

## 1. Introduction

Chronic hepatitis C virus (HCV) is the cause of the high proportion of cases of the chronic liver disease worldwide [1]. It is reported in many cohort studies that patients suffering from chronic hepatitis C (CHC) infection have more risk to hepatic morbidity and mortality, mainly due to the progression to liver cirrhosis [2,3] with a higher possibility of the development of systemic extra-hepatic metabolic complications [1,4]. Moreover, the high prevalence of insulin resistance (IR), diabetes, and fatty liver infiltration in individuals infected with HCV was also reported [5,6,7].

Kaddai et al. [8] stated that HCV is associated with insulin resistance. They also reported alteration in glucose metabolism during the early stages of chronic hepatitis C. Insulin resistance developed even earlier than HCV induced fibrosis in chronically infected HCV patients [9]. Reduction of viral load by the commonly used antiviral therapy is associated with marked improvement of glucose metabolism [10] and evidently reduced insulin resistance state [11] in such patients.

An increase in reactive oxygen species (ROS) and DNA damage precedes the development of a chronic hepatitis C complication. Even strong evidence indicates that both ROS and DNA damage is involved in carcinogenesis-related chronic hepatitis C [12]. Consequently, damaged DNA repair is an essential mechanism for cellular protection against cancer [13].

There are four major DNA repair pathways: mismatch repair, base excision repair (BER), nucleotide excision repair (NER), and double-strand break repair [14]. The main repair pathway is the base excision repair that protects cells against oxidative DNA lesions. The X-ray repair cross-complementing protein 1 *(XRCC1*) is an essential molecule in the pathway of base excision repair [15]. Additionally, genetic polymorphisms in genes encoding proteins involved in DNA repair might influence the subject’s susceptibility to cancer [16]. It has been reported that the human gene, X-ray repair cross-complementing group 1 (*XRCC1*), is considered an important candidate gene that influences susceptibility to HCC [16]. Several previous studies evaluate the possible association of the *XRCC1* gene single nucleotide polymorphisms (SNPs) (Arg280 histidine (His), arginine (Arg) 194 tryptophan (Trp), and Arg399 glutamine (Gln)) and risk of HCC [17]. But the association between c.1254C > T and c.1517G > C SNPs in *XRCC1* gene and susceptibility to HCC has not been analyzed.

Moreover, multiple studies have reported that oxidative stress might lead to impairment of insulin action [18,19,20]. However, the identity of the genes involved has not been fully elucidated.

The aim of the present study is to investigate the potential association of *XRCC1* genetic polymorphisms (c.1254 C > T and c.1517 G > C) with the development of insulin resistance in a sample of chronic hepatitis C Egyptian patients. Furthermore, this work analyzes the potential association of these two *XRCC1* gene SNPs with further development of complications (cirrhosis and hepatocellular carcinoma) in such patients.

## 2. Subjects and Methods

This study was carried out on 140 patients (98 males and 42 females) with chronic HCV infection with positive viremia, with or without HCC, aged 40–74 years old and recruited from the Tropical Medicine Department, Mansoura University, between June 2014 and May 2015. Seventy age- and sex-matched healthy subjects (with the age range 45–73 years) were also included in this work. All subjects have signed a written informed consent form before enrollment in the study. An approval was provided by the Institutional Review Board (IRB) of Mansoura Faculty of Medicine. All subjects had undergone detailed history taking, clinical assessment, and a Body Mass Index (BMI) calculation.

Patients were excluded if they have one of the following criteria: non-HCV related hepatocellular carcinoma, non-HCV related liver cirrhosis, hepatitis B or HIV infection, and diabetes mellitus or BMI > 30.

Liver function tests (ALT, AST, serum albumin, serum bilirubin, prothrombin time), creatinine, complete blood count, virology (HBS Ag, HCV Ab), RT-PCR for hepatitis C RNA for HCV Ab positive cases, AFP, were evaluated.

Furthermore, fasting blood glucose by a colorimetric method and fasting serum insulin with enzyme-linked immunosorbent assay (ELISA) technique were analyzed. The homeostasis model of IR (HOMA-IR) (for insulin resistance (IR) evaluation) was then calculated by the following equation:

HOMA-IR = fasting glucose (mmol/L) × fasting insulin (μIU/mL)/22.5 [21]. According to Wongwananuruk et al. [22], patients having HOMA IR > 2 were considered as having IR.

The HCC diagnosis was done according to the Barcelona-2000 conference on clinical management of hepatocellular carcinoma. Cirrhosis was diagnosed, on a clinical base, by laboratory tests, endoscopic evidence, sonography finding, and triphasic multislice computed tomography (CT) of the abdomen.

According to HOMA-IR results, patients (n = 140) were classified into two main groups: the IR group (n = 87) and the non-IR group (n = 53). The IR group was further sub-classified into the IR with HCC (IR-HCC) subgroup (n = 43) and the IR-cirrhotic subgroup (n = 44).

### 2.1. Sampling

After 8–12 h fasting, 5 mL of blood were collected from all subjects by venipuncture and divided into 2 mL of blood collected in a tube containing 200 μL ethylene diamine tetra-acetic acid (EDTA) to prevent blood coagulation and stored at −20 °C until extraction of DNA; the other 3 mL were collected in a plain tube with gel separator and this was left standing at room temperature for 15–30 min, then centrifuged at 3000 round per minute (rpm) for 10 min to obtain serum. The sera were used for fasting blood glucose levels assessment and the rest of the amount stored at −20 °C until assayed for fasting serum insulin.

### 2.2. Genotyping of XRCC1 Gene Polymorphisms

The DNA isolation was done using spin column DNA extraction kits (Gene-JET Whole Blood Genomic DNA Purification Mini Kit) according to the manufacturer’s instructions (Thermo-Scientific, Waltham, MA, USA, Cat. no #K0781). DNA quantification and purity were evaluated using Nanodrop (Thermo Scientific NanoDrop 2000 spectrophotometer, Waltham, MA, USA). The DNA concentration of samples was between 10 and 45 ng/μL. A260/A280 ratio represents the purity of the extracted DNA (~1.8 is generally accepted). The quality of DNA purified was assessed by separation on 1% agarose gel electrophoresis.

Genotyping of *XRCC1* SNPs (c.1254C > T and c.1517G > C) was analyzed by the polymerase chain reaction-restriction fragment length polymorphism (PCR-RFLP) method. The genotyping of c.1254C > T SNP was performed by the creation restriction site-PCR (CRS-PCR) method in which one primer contained a nucleotide mismatch, that makes the restriction enzyme used for discrimination of sequence variations [23]. The genotyping for c.1517G > C SNP was investigated by the PCR-RFLP technique according to Bi et al. [24].

PCR amplification was done using the following PCR reaction (25 µL): PCR master mix (12.5 µL), forward primer (2 µL of 10 pmol/µL), reverse primer (2 µL of 10pmol/µL), 5 µL extracted DNA template (10 ng/µL) and 3.5 µL double distilled water. The following primers sequences were used: for c.1254C > T SNP; Forward primer: 5′-GAGGAGGATGAGGCCTCTCACAC-3′and Reverse primer: 5′-TAAGGAGGGAGAGTGGGTGGGT-3′ while for c.1517G > C SNP; Forward primer: 5′-CAAGTCCCAGCTGAGAACTGAG-3′and Reverse primer: 5′-GCTGCTCTGCATGCTCACTC-3′. PCR amplification condition was initial denaturation (1 cycle) at 95 °C (5 min), 35 PCR cycles: denaturation at 95 °C (30 s), annealing at 59 °C (30 s), and extension at 72 °C (30 s), then final extension (1 cycle) at 72 °C (5 min). 2% agarose gel was used for separation of the DNA-PCR products, then the gel visualized by UV trans-illuminator (Model TUV-20, OWI Scientific, Inc. 800 242-5560, France). PCR product includes 2 variants [c.1254C > T (218 bp) and (c.1517G > C (247 bp)]. PCR product cleavage by *HpaII* restriction enzyme for c.1254C > T SNP and by Hae III restriction enzyme for c.1517G > C SNP was then performed.

The restriction enzyme digestion reaction contained these constituents in this sequence and volumes: 16 μL double distilled water, 3 μL 10 × Fast-Digest green buffer, 10 μL PCR product, and 1 μL of the restriction enzyme to get a net total amount 30 μL. Then the digestion mix was incubated at 37 °C in a heat block for 15 min. Finally, the restriction enzyme inactivation was done by heating at 65 °C for 5 min.

**Figure 1:**
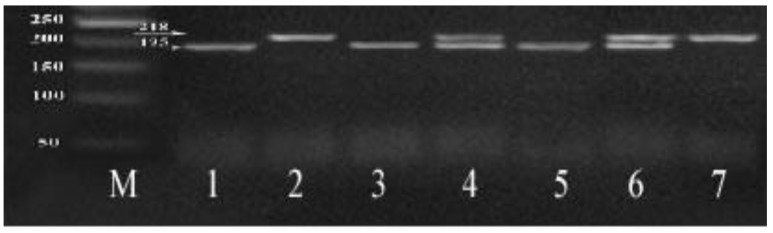
3% agarose gel for *c.1254C* > *T* digested products: 50 bp DNA ladder is in lane M, lanes 2, 7: contain one 218 bp band of T/T genotype, lanes 1, 3, 5: contain 195, 23 bp bands represent C/C genotype. Lanes 4, 6: contain 218, 195 bp bands and 23 bp (not seen) represent the C/T genotype.

**Figure 2:**
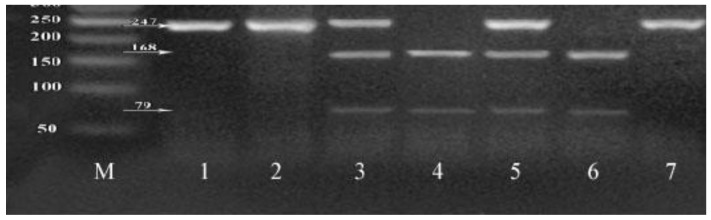
3% agarose gel for c.1517G > C digested product: 50 bp DNA ladder in lane M, lanes 1, 2, 7: contain one 247 bp band of G/G genotype, lanes 4, 6: contain 168, 79 bp bands represent C/C genotype. Lanes 3, 5: contain 247, 168, 79 bp bands represent the G/C genotype.

The amplified PCR products and products after restriction enzyme digestion were analyzed by 3% agarose gel electrophoresis. The PCR amplified products of c.1254 C > T, after digestion with restriction enzyme (*HpaII*), give three genotypes: CC (195 and 23 bp), CT (218, 195 and 23 bp), and TT (218bp) (Figure 1).The PCR amplified products of c.1517 G > C, after digestion with the restriction enzyme (*HaeIII*), give three genotypes: GG (247 bp), GA (247, 168 and 79 bp), and AA (168 and 79 bp) (Figure 2).

## 3. Statistical Analysis

Data were analyzed by the SPSS (Statistical package for social science) program version 17.0. Analysed data were presented as the Mean ± Standard Deviation (SD) and frequency (number-percent). The significance of difference was analyzed by the following statistical tests: analysis of variance (ANOVA) followed by post-hoc Tukey, and Mann-Whitney U test, or by Kruskal Wallis test and then the Mann-Whitney test for multiple comparisons.

The SNPs were tested for Hardy-Weinberg equilibrium and the genotypic and allelic disease association analysis was analyzed by the DeFinetti program. The frequencies of polymorphisms and genotype were evaluated by the gene counts. The X^2^ tests (2-by-2 tables) were done to calculate the significance for different genotype distributions and also odds ratio (OR) and 95% confidence interval were calculated to detect risk ratio. In all analyses, *p* values ≤ 0.05 were considered significant statistically.

## 4. Results

As a case-control study, a total of 210 subjects were enrolled (140 chronic HCV infection patients with or without HCC and 70 healthy controls). Chronic hepatitis C patients were further classified according to HOMA-IR into IR and non-IR groups. There was no significant difference between the three studied groups with regard to their age and gender distribution. The general criteria and biochemical parameters of the three studied groups are shown in Table 1. The IR group was further sub-classified into IR-HCC and IR-cirrhotic subgroups.

### 4.1. Genotyping of XRCC1 SNPs

The genotype frequencies of both c.1254 C > T and c.1517 G > C genetic polymorphisms corresponded to Hardy-Weinberg equilibrium. Table 2 and Table 3 show the allelic and genotypic frequencies of the two genetic variants. C-allele of c.1254 C > T and G-allele of c.1517 G > C genetic variants are the predominant alleles in all studied groups.

For the c.1254C > T (Table 2), the allelic analysis revealed that the percentage of individuals having the C allele frequency was 61% in IR group, 64% in non-IR group and 73% in healthy controls, while T allele was 39% in IR group, 36% of non-IR group and 27% of healthy controls, hence its frequency was significantly greater in IR patients than control subjects (P2 = 0.026). In addition, genotype analysis showed that the occurrence of the heterozygote genotype (CT) was significantly higher in the IR group than in the control group (P2 = 0.03). Also, the combined heterozygote and homozygote mutant (CT + TT) occurred more in the IR group (64.4%) than in the control group (45.7%) (P2 = 0.019). Meanwhile in the non-IR group, the mutant T allele, mutant homozygote genotype (TT), and heterozygote genotype (CT) were not significantly higher than in the control group. Furthermore, in chronic hepatitis C patients, there was a non-significant difference in allele or genotypes frequencies between the IR and the non-IR groups.

Similarly, as for c.1517G > C, the allelic analysis revealed that the percentage of individuals having the G allele frequency was 67% in the IR group and 77% in healthy controls while C allele was 33% of the IR group and 23% of healthy controls hence its frequency was significantly higher in IR than controls (P2 = 0.04). Also, genotype analysis shows that the occurrence of the heterozygote genotype (GC) was significantly greater in IR patients than in control subjects (P2 = 0.025). Also, the combined heterozygote and homozygote mutant (GC + CC) occurred significantly more in the IR group (58.6%) than in control group (40.0%) (P2 = 0.02). Furthermore, in the non-IR group, the mutant C allele, mutant homozygote genotype (CC) and heterozygote genotype (GC) were significantly greater than the control group (P3 = 0.001, P3 = 0.008, P3 = 0.005 respectively). Meanwhile in chronic hepatitis C patients, there was a non-significant difference in allele or genotypes frequencies between the IR and non-IR groups (Table 2).

Haplotyping analysis (Table 4) revealed that in all 210 studied subjects, only one case with non-IR chronic HCV infection was carrying the combined mutant genotypes (TTCC). The occurrence of the haplotype heterozygote of both variants of the gene (CTGC) and the combination of a homozygous mutant of c.1254C > T SNP and heterozygote of c.1517G > C SNP (TTGC) occurred significantly more in the IR group than in control group (P2 = 0.007, P2 = 0.006 respectively). However, only the haplotype (CTGG) was significantly higher in the IR group than in the non-IR group (P1 = 0.003). No other haplotypes were significantly different in different studied groups.

### 4.2. Association between XRCC1 SNPs and IR Risk in Chronic HCV Patients

With regard to the c.1254C > T SNP, the combined heterozygote and homozygote mutant (CT + TT) genotype or carrying mutant T allele is associated with increased risk for developing IR than controls (OR = 2.14, 95% CI 1.13–4.08, P2 = 0.019, OR = 1.7, 95% CI 1.06–2.78, P2 = 0.026, respectively). However, no significant difference for carrying mutant genotype (CT or TT) or mutant T allele in comparing IR and non-IR HCV patients (P1 > 0.05), indicating that c.1254C > T SNP may not be related to having IR in chronic HCV infection. Meanwhile for c.1517G > C SNP, carrying mutant genotype (GC or CC) or mutant C allele is associated with HCV infection either IR or non-IR patients (both P2 and P3 ≤ 0.05) except for mutant CC genotype in the IR group. There is no significant difference for carrying the mutant genotype (GC or CC) or the mutant C allele in comparing IR and non-IR HCV patients (P1 > 0.05). These results indicate that for c.1517G > C SNP, carrying mutant genotype (GC or CC,) or mutant C alleles may be associated with HCV infection but not related to developing IR in these patients (Table 2).

For the association between *XRCC1* SNPs and HCC risk in IR patients, Table 3 also represents the association of *XRCC1* SNPs and the risk for HCC. With regard to the c.1254C > T, in chronic HCV with IR patients, there were statistically significant increased risk of HCC with regard to the homozygote comparison (TT versus CC: OR = 9.09, 95% CI 1.68–49.12, P1 = 0.007), and allele comparison (T versus C: OR = 2.27, 95% CI 1.2–4.2, P1 = 0.009). Furthermore, the risk of developing HCC in IR patients compared to healthy subjects is statistically increased with carrying either mutant T allele or mutant genotype (CT or TT).

However, for c.1517G > C, in chronic HCV with IR patients, no significantly increased risk of HCC was present with carrying either the mutant C allele or mutant genotype (GC or CC) (P1 > 0.05). Meanwhile, the presence of the mutant C allele significantly increases the risk of HCC compared to control subjects (OR = 2.00, 95% CI 1.11–3.6, P2 = 0.02).

## 5. Discussion

The hepatitis C virus (HCV) infection is the main reason for chronic hepatitis. Chronic hepatitis C usually progresses to liver cirrhosis and may be complicated by hepatocellular carcinoma [25]. Metabolic syndrome is a common metabolic disorder found in HCV infected patients. It is presented by features like insulin resistance that may lead to diabetes mellitus type 2, nonalcoholic fatty liver disease, disorders of the cardiovascular system, and several types of cancers, including hepatocellular carcinoma [26,27].

The first objective of our study is to investigate the possible association of *XRCC1* genetic polymorphisms (c.1254 C > T and c.1517 G > C) with the development of insulin resistance in a sample of chronic hepatitis C Egyptian patients.

Regarding insulin resistance, the current study demonstrated that in HCV patient group fasting blood glucose level, fasting insulin level and HOMA- IR were significantly higher than the control group (data not presented). This finding is consistent with Hui et al. [28] and Abd el-star et al. [29]. Imazeki and his team reported that diabetes mellitus type 2 and/or insulin resistance were independently more prevalent among chronic HCV patients in comparison to control groups while in HBV and HCV-eradicated patients this association was confirmed only with the presence of some confounding factors. Insulin resistance was improved by antiviral treatment and this reveals a strong mechanistic relationship between HCV infections and diabetes mellitus type 2 incidence [30]. Furthermore, Mohamed et al. [31] concluded in their study that IR may be induced by HCV genotype-4 infection regardless of the severity of liver disease and that IR effects begin at an early phase of HCV infection and accelerate the progression of hepatic fibrosis and HCC development [31].

A significant difference in AFP levels between non-IR and IR patients is detected in this work. This may be also due to the inclusion of HCC patients in both groups with an unequal percentage. The relationship between serum AFP levels and insulin resistance seems to be unclear. The results of Xu and other researchers suggested that there was a significant association of serum AFP levels and fatty liver disease but as a cofactor, not as an independent factor [32]. Chen and his team proposed that hepatocyte necrosis followed by hepatic regeneration especially biliary epithelial cells may be the cause for increase serum AFP level [33]. Also in 2013, Kawaguchi’s study suggested that there is an association between whole-body IR and increased serum AFP level in HCV patients using whole-body insulin sensitivity index (WBISI) but did not reveal a linkage between elevated AFP level and the HOMA-IR [34]. These results proposed that an association between IR and increased AFP levels may be due to an HCV infected liver as well as obesity or other metabolic conditions, as systemic IR develops in many organs simultaneously (including liver, skeletal muscle, and adipose tissue).

Regarding c.1254C > T polymorphism and IR, there was a significantly higher frequency of heterozygote genotype CT, combined (CT + TT) and T allele in the IR group more than the control group. These findings suggest that there might be a relationship between the *XRCC1* gene polymorphism and the development of IR. In comparison between IR group and non-IR group, there was a non-significant difference in genotypic and allelic frequencies. This may suggest that there might be no association between *XRCC1* polymorphism and the role of HCV infection in the development of IR.

Regarding c.1517G > C and IR, when genotype and allelic frequencies were compared in the IR group, the non-IR group, and healthy controls, there was a significantly higher frequency of heterozygote genotype GC, combined (GC + CC) and C allele in either non-IR or IR groups more than the control group. This may be due to an involvement of HCC patients in both patient groups with an unequal percentage. In a comparison between the IR group and the non-IR group, there was a non-significant difference in genotypic and allelic frequencies.

To the best of our knowledge, no other studies were found supporting or contradicting this suggestion about the relationship between the *XRCC1* gene and development of IR and to the best of our knowledge, this is the first report for this possible relationship, which needs further investigation on a larger number of populations.

The *XRCC1* protein is an essential molecule in the multistep base excision repair pathway, and its gene is the first isolated mammalian gene that influences cell sensitivity for ionizing radiation [35]. Mutations of the *XRCC1* gene might increase cancer risk through impairment of *XRCC1* interaction with further enzymatic proteins and so induce alterations in DNA repair activity [36,37], and this may subsequently induce carcinogenesis, including tumors of head and neck, lung, breast, esophagus, and multiple other tumors [38,39].

The *XRCC1* gene is an important candidate gene for HCC, and the association between several SNPs in the *XRCC1* gene (Arg194Trp, Arg280His, and Arg399Gln) with the risk of HCC have been assessed in recent years [16,17,39,40,41]. However, the results are conflicting due to the diversity in cancer types, the source of cases, ethnicities, and sample size. Additionally, several studies have demonstrated the impairment in insulin action by oxidative stress [20,42]. HCV infection causes oxidative stress leading to impairment of insulin action, steatosis, fibrosis, apoptosis, gene expression alteration, and HCC [43]. But genetic factors affecting the development of insulin resistance in HCV patients have not yet been completely investigated. Therefore, the second objective of our study was to investigate the XRCC1 gene variants influencing the susceptibility of HCC related to HCV infection in the Egyptian population and to link it to the state of insulin resistance.

Analysis of c.1254C > T *XRCC1* gene polymorphism at the level of IR subgroups revealed a significantly higher frequency of heterozygote genotype CT, mutant genotype TT, combined genotype (CT + TT) and T allele in the IR-HCC group more than the control group. Additionally, for c.1517G > C *XRCC1* gene polymorphism, at the level of IR subgroups, the comparison between IR-HCC group, IR-cirrhotic, and the control group showed a significantly higher frequency of heterozygote genotype GC, combined (GC + CC) and C allele in IR-HCC group more than the control.

Therefore, these findings suggest that the T-allele of c.1254C > T and the C-allele of c.1517G > C genetic variants may be related to the susceptibility of HCC and that there might be a relationship between XRCC1 gene polymorphism and development of HCC in patients with insulin resistance. These results agree with Bi and his team in their study on the Chinese Han Population in 2013, who reported that HCC risk increases significantly with T allele and TT genotype c.1254C > T variant and C allele and CC genotype c.1517G > C variant of XRCC1 gene [24].

This study has some limitations. Firstly, the small number of cases may reduce the statistical power to find the difference between groups. Secondly, the undefined cutoff value for IR by HOMA-IR which ranges from 1.5 to 3 [44] and leads to possible misinterpretation of results and contradiction between studies in this aspect. In this study, we considered patients with HOMA-IR > 2 have IR [22]. Thirdly, the non-exclusion of overweight patients (BMI = 25.0–29.9 kg/m^2^), as we only exclude obese patients (BMI ≥ 30 kg/m^2^), was a limitation. Finally, further investigations and studies on greater population samples are necessary to confirm our results or to contradict and to define the relationship between this gene polymorphism and HCV induced HCC, and whether it is actually related to IR in these patients.

## 6. Conclusions

The current study could conclude that HCV related chronic liver disease is associated with IR, though that could be considered as a coincident event with the course of the disease progression. The T-allele of c.1254C > T and C-allele of c.1517G > C genetic variants of the *XRCC1* gene might be associated with increased risk for IR development in HCV infected patients. Moreover, the T-allele of c.1254C > T and C-allele of c.1517G > C genetic variants might increase the susceptibility of HCC. As the T allele of c.1254C > T was significantly higher in IR-HCC patients than IR-cirrhotic, HCV infected patients with IR and carrying T allele may be more liable to develop HCC. Finally, the *XRCC1* gene polymorphism could be considered as a possible molecular candidate for IR susceptibility in chronic HCV patients, which needs further investigation.

## Figures and Tables

**Table 1:** Demographic data, AFP, BMI, and homeostasis model of IR (HOMA-IR) in insulin resistant (IR), non-insulin resistant (non-IR) and control groups.

	Control		Non-IR		IR		P	P1	P2	P3
Age	56.6	7.2	58.7	6.7	56.9	5.6	0.2			
Female	22	31.4%	16	30.2%	26	29.9%	0.97			
Male	48	68.6%	37	69.8%	61	70.1%				
BMI	25.11	2.77	26.3	4.64	26.63	5.1	0.09			
AFP	6.20	2.60–11.60	19.20	2.70–4233.60	33.50	6.00–5000.00	<0.001	0.06	<0.001	0.015
HOMA-IR	1.5	2.0–0.3	0.9	2.0–0.2	3.4	13.8–2.1	<0.001	0.09	<0.001	<0.001

Data expressed either as mean ± SD or median (range). SD: standard deviation; P: Probability. Test used: One-way ANOVA followed by post-hoc Tukey for data expressed as mean ± SD and Kruskalwallis test followed by Mann-Whitney for data expressed as median and range. P1: Significance between Control and Non-IR groups. P2: Significance between Control and IR groups. P3: Significance between Non-IR and IR groups.

**Table 2:** Genotype and allele analysis of XRCC1 gene in IR, Non-IR and control groups.

Genotype and Allele Analysis of c.1254 C > T Polymorphism of *XRCC1* Gene										
		All Patients		Control Group (70) No (%)	P1	OR1 (CI 95%)	P2	OR2 (CI 95%)	P3	OR3 (CI 95%)
		IR (87) No (%)	Non-IR (53) No (%)							
**Genotypes**	**CC**	31 (35.6%)	24 (45.3%)	38 (54.3%)	-	1 (Ref)	-	1 (Ref)	-	1 (Ref)
	**CT**	44 (50.6%)	20 (37.7%)	26 (37.1%)	0.16	1.7 (0.8–3.6)	0.03	2.07 (1.05–4.09)	0.6	1.2 (0.56–2.6)
	**TT**	12 (13.8%)	9 (17.0%)	6 (8.6%)	0.95	1.03 (0.37–2.8)	0.1	2.45 (0.8–7.3)	0.13	2.37 (0.75–7.5)
	**CT + TT**	56 (64.4%)	29 (54.7%)	32 (45.7%)	0.25	1.5 (0.7–3.00)	0.019	2.14 (1.13–4.08)	0.3	1.4 (0.7–2.9)
**Alleles**	**C**	106 (61.0%)	68 (64.0%)	102 (73.0%)	-	1 (Ref)	-	1 (Ref)	-	1 (Ref)
	**T**	68 (39.0%)	38 (36.0%)	38 (27.0%)	0.58	1.14 (0.7–1.9)	0.026	1.7 (1.06–2.78)	0.14	1.5 (0.87–2.58)
**Genotype and Allele Analysis of c.1517 G > C Polymorphism of *XRCC1* Gene**										
**Genotypes**	**GG**	36 (41.4%)	16 (30.2%)	42 (60.0%)	-	1 (Ref)	-	1 (Ref)	-	1 (Ref)
	**GC**	44 (50.6%)	28 (52.8%)	24 (34.3%)	0.35	0.69 (0.1–1.09)	0.025	2.1 (1.097–4.16)	0.005	3.06 (1.4–6.76)
	**CC**	7 (8.0%)	9 (17.0%)	4 (5.7%)	0.065	0.34 (0.37–2.8)	0.3	2.04 (0.55–7.5)	0.008	5.9 (1.6–21.9)
	**GC + CC**	51 (58.6%)	37 (69.8%)	28 (40.0%)	0.18	0.6 (0.3–1.26)	0.02	2.14 (1.13–4.08)	0.001	3.5 (1.6–7.4)
**Alleles**	**G**	116 (67.0%)	60 (57.0%)	108 (77.0%)	-	1 (Ref)	-	1 (Ref)	-	1 (Ref)
	**C**	58 (33.0%)	46 (43.0%)	32 (23.0%)	0.09	0.65 (0.4–1.07)	0.04	1.68 (1.02–2.8)	0.001	2.55 (1.5–4.48)

P: Probability. OR: Odds ratio. CI: confidence interval. P1: significance between INR group and non-INR group. P2: significance between INR group and control group. P3: significance between non-INR group and control group.

**Table 3:** Genotype and allele analysis of XRCC1 gene in IR-cirrhotic and IR-HCC and control groups.

Genotype and Allele Analysis of c.1254 C > T Polymorphism of *XRCC1* Gene										
		IR		Control Group (70) No (%)	P1	OR1 (CI 95%)	P2	OR2 (CI 95%)	P3	OR3 (CI 95%)
		IR–HCC (43) No (%)	IR-Cirrhotic (44) No (%)							
**Genotypes**	**CC**	11 (25.6%)	20 (63.6%)	38 (54.3%)	-	1 (Ref)	-	1 (Ref)	-	1 (Ref)
	**CT**	22 (51.2%)	22 (33.3%)	26 (37.1%)	0.2	1.8 (0.7–4.67)	0.015	2.9 (1.2–7.04)	0.2	1.6 (0.7–3.5)
	**TT**	10 (23.3%)	2 (3.0%)	6 (8.6%)	0.007	9.09 (1.68–49.12)	0.003	5.7 (1.7–19.4)	0.7	0.6 (0.11–3.4)
	**CT + TT**	32 (74.5%)	24 (36.3%)	32 (45.7%)	0.053	2.4 (0.97–6.00)	0.003	3.45 (1.5–7.9)	0.35	1.4 (0.66–3.04)
**Alleles**	**C**	44 (51.0%)	62 (70.0%)	102 (73.0%)	-	1 (Ref)	-	1 (Ref)	-	1 (Ref)
	**T**	42 (49.0%)	26 (30.0%)	38 (27.0%)	0.009	2.27 (1.2–4.2)	0.001	2.56 (1.45–4.5)	0.7	1.12 (0.6–2.03)
**Genotype and Allele Analysis of c.1517 G > C Polymorphism of *XRCC1* Gene**										
**Genotypes**	**GG**	14 (32.6%)	22 (50.0%)	42 (60.0%)	-	1 (Ref)	-	1 (Ref)	-	1 (Ref)
	**GC**	26 (60.5%)	18 (40.9%)	24 (34.3%)	0.07	2.27 (0.9–5.58)	0.004	3.25 (1.4–7.4)	0.37	1.4 (0.6–3.2)
	**CC**	3 (7.0%)	4 (9.1%)	4 (5.7%)	1.00	1.17 (0.22–6.07)	0.37	2.25 (0.44–11.3)	0.44	1.9 (0.4–8.4)
	**GC + CC**	32 (74.5%)	24 (36.3%)	28 (40.0%)	0.087	2.095 (0.9–4.9)	0.001	4.00 (1.8–8.9)	0.19	1.6 (0.77–3.5)
**Alleles**	**G**	54 (63.0%)	62 (70.0%)	108 (77.0%)	-	1 (Ref)	-	1 (Ref)	-	1 (Ref)
	**C**	32 (37.0%)	26 (30.0%)	32 (23.0%)	0.28	1.4 (0.75–2.66)	0.02	2.00 (1.11–3.6)	0.27	1.4 (0.77–2.6)

P: Probability. OR: Odds ratio. CI: confidence interval. P1: significance between IR-HCC group and IR-cirrhotic group. P2: significance between IR-HCC and control group. P3: significance between IR-cirrhotic group and control group.

**Table 4:** Haplotyping analysis of c.1254 C > T and c.1517 G > C of XRCC1 gene polymorphisms in IR, non-IR and control groups.

	IR (87) No (%)	Non-IR (53) No (%)	Control Group (70) No (%)	P1	OR1 (CI 95%)	P2	OR2 (CI 95%)	P3	OR3 (CI 95%)
**CCGG**	10 (11.5%)	10 (18.9%)	20 (28.6%)	-	1 (Ref)	-	1 (Ref)	-	1 (Ref)
**CCGC**	16 (18.4%)	8 (15.1%)	14 (20.0%)	0.26	2.0 (0.6–6.77)	0.11	2.28 (0.8–6.5)	0.8	1.14 (0.36–3.6)
**CCCC**	5 (5.7%)	6 (11.3%)	4 (5.7%)	0.8	0.83 (0.19–3.6)	0.22	2.5 (0.5–11.4)	0.16	3.00 (0.69–13.11)
**CTGG**	24 (27.6%)	3 (5.7%)	18 (25.7%)	0.003	8.00 (1.8–35.36)	0.046	2.66 (1.006–7.07)	0.19	0.33 (0.08–1.4)
**CTGC**	18 (20.7%)	15 (28.3%)	8 (11.4%)	0.75	1.2 (0.39–3.65)	0.007	4.5 (1.45–13.88)	0.02	3.75 (1.19–11.8)
**CTCC**	2 (2.3%)	2 (3.8%)	0 (0%)	1.00	1.00 (0.11–8.55)	0.13	-	0.13	-
**TTGG**	2 (2.3%)	3 (5.7%)	4 (5.7%)	0.69	0.66 (0.09–4.88)	1.00	1.00 (0.16–6.4)	0.68	1.5 (0.28–8.04)
**TTGC**	10 (11.5%)	5 (9.4%)	2 (2.9%)	0.3	2.00 (0.5–7.99)	0.006	10.00 (1.8–54.6)	0.095	5.00 (0.8–30.46)
**TTCC**	0 (0%)	1 (1.9%)	0 (0%)	0.3	-	-	-	0.35	-

P: Probability. OR: Odds ratio. CI: confidence interval. P1: significance between IR group and non-IR group. P2: significance between IR group and control group. P3: significance between non-IR group and control group.
